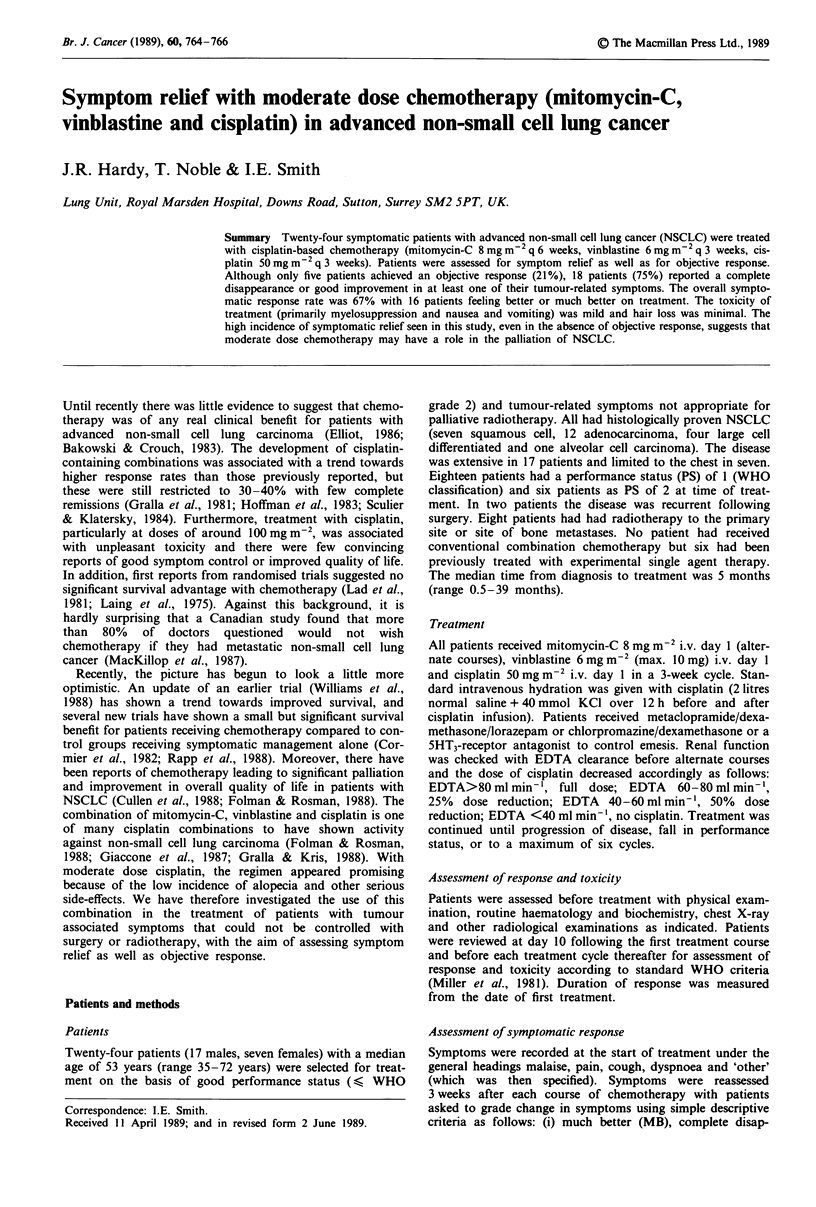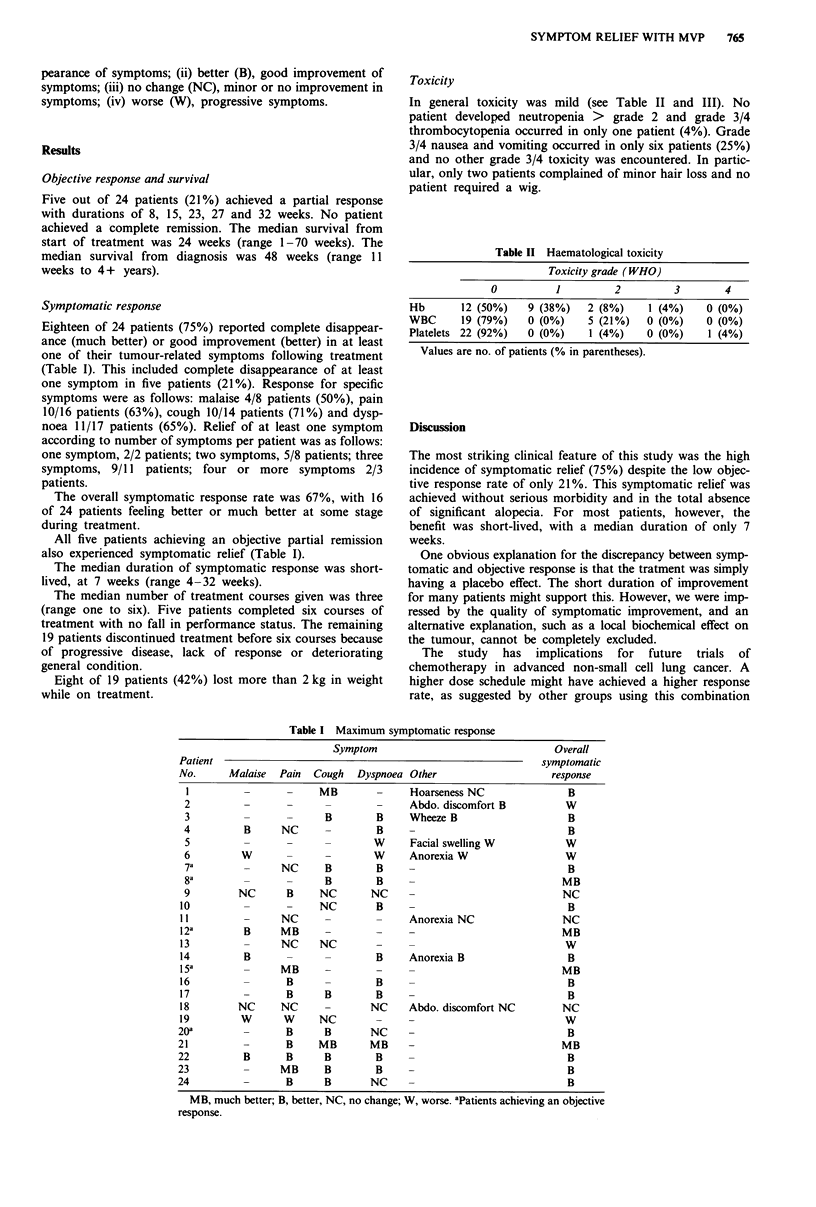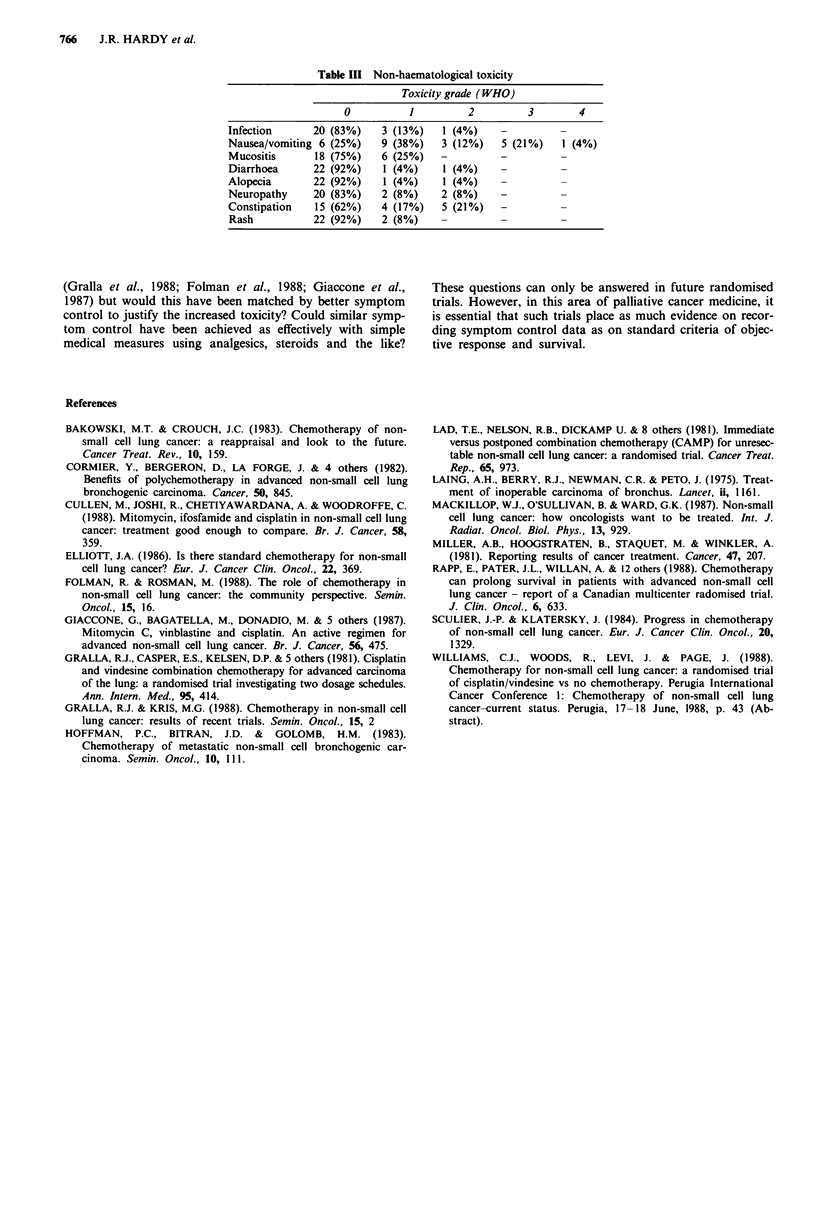# Symptom relief with moderate dose chemotherapy (mitomycin-C, vinblastine and cisplatin) in advanced non-small cell lung cancer.

**DOI:** 10.1038/bjc.1989.355

**Published:** 1989-11

**Authors:** J. R. Hardy, T. Noble, I. E. Smith

**Affiliations:** Lung Unit, Royal Marsden Hospital, Sutton, Surrey, UK.

## Abstract

Twenty-four symptomatic patients with advanced non-small cell lung cancer (NSCLC) were treated with cisplatin-based chemotherapy (mitomycin-C 8 mg m-2 q 6 weeks, vinblastine 6 mg m-2 q 3 weeks, cisplatin 50 mg m-2 q 3 weeks). Patients were assessed for symptom relief as well as for objective response. Although only five patients achieved an objective response (21%), 18 patients (75%) reported a complete disappearance or good improvement in at least one of their tumour-related symptoms. The overall symptomatic response rate was 67% with 16 patients feeling better or much better on treatment. The toxicity of treatment (primarily myelosuppression and nausea and vomiting) was mild and hair loss was minimal. The high incidence of symptomatic relief seen in this study, even in the absence of objective response, suggests that moderate dose chemotherapy may have a role in the palliation of NSCLC.


Br. J. Cancer (1989), 60, 764-766 © The Macmillan Press Ltd., 1989

### Symptom relief with moderate dose chemotherapy (mitomycin-C, vinblastine and cisplatin) in advanced non-small cell lung cancer

J.R. Hardy, T. Noble & I.E. Smith

*Lung Unit, Royal Marsden Hospital, Downs Road, Sutton, Surrey SM2 5PT, UK.*

**Summary** Twenty-four symptomatic patients with advanced non-small cell lung cancer (NSCLC) were treated with cisplatin-based chemotherapy (mitomycin-C 8 mg m$^{-2}$ q 6 weeks, vinblastine 6 mg m$^{-2}$ q 3 weeks, cisplatin 50 mg m$^{-2}$ q 3 weeks). Patients were assessed for symptom relief as well as for objective response. Although only five patients achieved an objective response (21%), 18 patients (75%) reported a complete disappearance or good improvement in at least one of their tumour-related symptoms. The overall symptomatic response rate was 67% with 16 patients feeling better or much better on treatment. The toxicity of treatment (primarily myelosuppression and nausea and vomiting) was mild and hair loss was minimal. The high incidence of symptomatic relief seen in this study, even in the absence of objective response, suggests that moderate dose chemotherapy may have a role in the palliation of NSCLC.

Until recently there was little evidence to suggest that chemotherapy was of any real clinical benefit for patients with advanced non-small cell lung carcinoma (Elliot, 1986; Bakowski & Crouch, 1983). The development of cisplatin-containing combinations was associated with a trend towards higher response rates than those previously reported, but these were still restricted to 30–40% with few complete remissions (Gralla *et al.*, 1981; Hoffman *et al.*, 1983; Sculier & Klatersky, 1984). Furthermore, treatment with cisplatin, particularly at doses of around 100 mg m$^{-2}$, was associated with unpleasant toxicity and there were few convincing reports of good symptom control or improved quality of life. In addition, first reports from randomised trials suggested no significant survival advantage with chemotherapy (Lad *et al.*, 1981; Laing *et al.*, 1975). Against this background, it is hardly surprising that a Canadian study found that more than 80% of doctors questioned would not wish chemotherapy if they had metastatic non-small cell lung cancer (MacKillop *et al.*, 1987).

Recently, the picture has begun to look a little more optimistic. An update of an earlier trial (Williams *et al.*, 1988) has shown a trend towards improved survival, and several new trials have shown a small but significant survival benefit for patients receiving chemotherapy compared to control groups receiving symptomatic management alone (Cormier *et al.*, 1982; Rapp *et al.*, 1988). Moreover, there have been reports of chemotherapy leading to significant palliation and improvement in overall quality of life in patients with NSCLC (Cullen *et al.*, 1988; Folman & Rosman, 1988). The combination of mitomycin-C, vinblastine and cisplatin is one of many cisplatin combinations to have shown activity against non-small cell lung carcinoma (Folman & Rosman, 1988; Giaccone *et al.*, 1987; Gralla & Kris, 1988). With moderate dose cisplatin, the regimen appeared promising because of the low incidence of alopecia and other serious side-effects. We have therefore investigated the use of this combination in the treatment of patients with tumour associated symptoms that could not be controlled with surgery or radiotherapy, with the aim of assessing symptom relief as well as objective response.

### Patients and methods

### *Patients*

Twenty-four patients (17 males, seven females) with a median age of 53 years (range 35–72 years) were selected for treatment on the basis of good performance status ($\leqslant$ WHO grade 2) and tumour-related symptoms not appropriate for palliative radiotherapy. All had histologically proven NSCLC (seven squamous cell, 12 adenocarcinoma, four large cell differentiated and one alveolar cell carcinoma). The disease was extensive in 17 patients and limited to the chest in seven. Eighteen patients had a performance status (PS) of 1 (WHO classification) and six patients as PS of 2 at time of treatment. In two patients the disease was recurrent following surgery. Eight patients had had radiotherapy to the primary site or site of bone metastases. No patient had received conventional combination chemotherapy but six had been previously treated with experimental single agent therapy. The median time from diagnosis to treatment was 5 months (range 0.5–39 months).

### *Treatment*

All patients received mitomycin-C 8 mg m$^{-2}$ i.v. day 1 (alternate courses), vinblastine 6 mg m$^{-2}$ (max. 10 mg) i.v. day 1 and cisplatin 50 mg m$^{-2}$ i.v. day 1 in a 3-week cycle. Standard intravenous hydration was given with cisplatin (2 litres normal saline + 40 mmol KCl over 12 h before and after cisplatin infusion). Patients received metaclopramide/dexamethasone/lorazepam or chlorpromazine/dexamethasone or a $5HT_3$-receptor antagonist to control emesis. Renal function was checked with EDTA clearance before alternate courses and the dose of cisplatin decreased accordingly as follows: EDTA>80 ml min$^{-1}$, full dose; EDTA 60–80 ml min$^{-1}$, 25% dose reduction; EDTA 40–60 ml min$^{-1}$, 50% dose reduction; EDTA <40 ml min$^{-1}$, no cisplatin. Treatment was continued until progression of disease, fall in performance status, or to a maximum of six cycles.

### *Assessment of response and toxicity*

Patients were assessed before treatment with physical examination, routine haematology and biochemistry, chest X-ray and other radiological examinations as indicated. Patients were reviewed at day 10 following the first treatment course and before each treatment cycle thereafter for assessment of response and toxicity according to standard WHO criteria (Miller *et al.*, 1981). Duration of response was measured from the date of first treatment.

### *Assessment of symptomatic response*

Symptoms were recorded at the start of treatment under the general headings malaise, pain, cough, dyspnoea and 'other' (which was then specified). Symptoms were reassessed 3 weeks after each course of chemotherapy with patients asked to grade change in symptoms using simple descriptive criteria as follows: (i) much better (MB), complete disap-

Correspondence: I.E. Smith.
Received 11 April 1989; and in revised form 2 June 1989.

pearance of symptoms; (ii) better (B), good improvement of symptoms; (iii) no change (NC), minor or no improvement in symptoms; (iv) worse (W), progressive symptoms.

### Results

*Objective response and survival*

Five out of 24 patients (21%) achieved a partial response with durations of 8, 15, 23, 27 and 32 weeks. No patient achieved a complete remission. The median survival from start of treatment was 24 weeks (range 1–70 weeks). The median survival from diagnosis was 48 weeks (range 11 weeks to 4+ years).

*Symptomatic response*

Eighteen of 24 patients (75%) reported complete disappearance (much better) or good improvement (better) in at least one of their tumour-related symptoms following treatment (Table I). This included complete disappearance of at least one symptom in five patients (21%). Response for specific symptoms were as follows: malaise 4/8 patients (50%), pain 10/16 patients (63%), cough 10/14 patients (71%) and dyspnoea 11/17 patients (65%). Relief of at least one symptom according to number of symptoms per patient was as follows: one symptom, 2/2 patients; two symptoms, 5/8 patients; three symptoms, 9/11 patients; four or more symptoms 2/3 patients.

The overall symptomatic response rate was 67%, with 16 of 24 patients feeling better or much better at some stage during treatment.

All five patients achieving an objective partial remission also experienced symptomatic relief (Table I).

The median duration of symptomatic response was short-lived, at 7 weeks (range 4–32 weeks).

The median number of treatment courses given was three (range one to six). Five patients completed six courses of treatment with no fall in performance status. The remaining 19 patients discontinued treatment before six courses because of progressive disease, lack of response or deteriorating general condition.

Eight of 19 patients (42%) lost more than 2 kg in weight while on treatment.

*Toxicity*

In general toxicity was mild (see Table II and III). No patient developed neutropenia > grade 2 and grade 3/4 thrombocytopenia occurred in only one patient (4%). Grade 3/4 nausea and vomiting occurred in only six patients (25%) and no other grade 3/4 toxicity was encountered. In particular, only two patients complained of minor hair loss and no patient required a wig.

**Table II** Haematological toxicity

| | Toxicity grade (WHO) | | | | |
|---|---|---|---|---|---|
| | 0 | 1 | 2 | 3 | 4 |
| Hb | 12 (50%) | 9 (38%) | 2 (8%) | 1 (4%) | 0 (0%) |
| WBC | 19 (79%) | 0 (0%) | 5 (21%) | 0 (0%) | 0 (0%) |
| Platelets | 22 (92%) | 0 (0%) | 1 (4%) | 0 (0%) | 1 (4%) |

Values are no. of patients (% in parentheses).

### Discussion

The most striking clinical feature of this study was the high incidence of symptomatic relief (75%) despite the low objective response rate of only 21%. This symptomatic relief was achieved without serious morbidity and in the total absence of significant alopecia. For most patients, however, the benefit was short-lived, with a median duration of only 7 weeks.

One obvious explanation for the discrepancy between symptomatic and objective response is that the tratment was simply having a placebo effect. The short duration of improvement for many patients might support this. However, we were impressed by the quality of symptomatic improvement, and an alternative explanation, such as a local biochemical effect on the tumour, cannot be completely excluded.

The study has implications for future trials of chemotherapy in advanced non-small cell lung cancer. A higher dose schedule might have achieved a higher response rate, as suggested by other groups using this combination

**Table I** Maximum symptomatic response

| Patient No. | Symptom: Malaise | Pain | Cough | Dyspnoea | Other | Overall symptomatic response |
|---|---|---|---|---|---|---|
| 1 | – | – | MB | – | Hoarseness NC | B |
| 2 | – | – | – | – | Abdo. discomfort B | W |
| 3 | – | – | B | B | Wheeze B | B |
| 4 | B | NC | – | B | – | B |
| 5 | – | – | – | W | Facial swelling W | W |
| 6 | W | – | – | W | Anorexia W | W |
| 7[a] | – | NC | B | B | – | B |
| 8[a] | – | – | B | B | – | MB |
| 9 | NC | B | NC | NC | – | NC |
| 10 | – | – | NC | B | – | B |
| 11 | – | NC | – | – | Anorexia NC | NC |
| 12[a] | B | MB | – | – | – | MB |
| 13 | – | NC | NC | – | – | W |
| 14 | B | – | – | B | Anorexia B | B |
| 15[a] | – | MB | – | – | – | MB |
| 16 | – | B | – | B | – | B |
| 17 | – | B | B | B | – | B |
| 18 | NC | NC | – | NC | Abdo. discomfort NC | NC |
| 19 | W | W | NC | – | – | W |
| 20[a] | – | B | B | NC | – | B |
| 21 | – | B | MB | MB | – | MB |
| 22 | B | B | B | B | – | B |
| 23 | – | MB | B | B | – | B |
| 24 | – | B | B | NC | – | B |

MB, much better; B, better, NC, no change; W, worse. [a]Patients achieving an objective response.

**Table III** Non-haematological toxicity

| | Toxicity grade (WHO) | | | | |
|---|---|---|---|---|---|
| | 0 | 1 | 2 | 3 | 4 |
| Infection | 20 (83%) | 3 (13%) | 1 (4%) | – | – |
| Nausea/vomiting | 6 (25%) | 9 (38%) | 3 (12%) | 5 (21%) | 1 (4%) |
| Mucositis | 18 (75%) | 6 (25%) | – | – | – |
| Diarrhoea | 22 (92%) | 1 (4%) | 1 (4%) | – | – |
| Alopecia | 22 (92%) | 1 (4%) | 1 (4%) | – | – |
| Neuropathy | 20 (83%) | 2 (8%) | 2 (8%) | – | – |
| Constipation | 15 (62%) | 4 (17%) | 5 (21%) | – | – |
| Rash | 22 (92%) | 2 (8%) | – | – | – |

(Gralla *et al.*, 1988; Folman *et al.*, 1988; Giaccone *et al.*, 1987) but would this have been matched by better symptom control to justify the increased toxicity? Could similar symptom control have been achieved as effectively with simple medical measures using analgesics, steroids and the like? These questions can only be answered in future randomised trials. However, in this area of palliative cancer medicine, it is essential that such trials place as much evidence on recording symptom control data as on standard criteria of objective response and survival.

### References

BAKOWSKI, M.T. & CROUCH, J.C. (1983). Chemotherapy of non-small cell lung cancer: a reappraisal and look to the future. *Cancer Treat. Rev.*, **10**, 159.

CORMIER, Y., BERGERON, D., LA FORGE, J. & 4 others (1982). Benefits of polychemotherapy in advanced non-small cell lung bronchogenic carcinoma. *Cancer*, **50**, 845.

CULLEN, M., JOSHI, R., CHETIYAWARDANA, A. & WOODROFFE, C. (1988). Mitomycin, ifosfamide and cisplatin in non-small cell lung cancer: treatment good enough to compare. *Br. J. Cancer*, **58**, 359.

ELLIOTT, J.A. (1986). Is there standard chemotherapy for non-small cell lung cancer? *Eur. J. Cancer Clin. Oncol.*, **22**, 369.

FOLMAN, R. & ROSMAN, M. (1988). The role of chemotherapy in non-small cell lung cancer: the community perspective. *Semin. Oncol.*, **15**, 16.

GIACCONE, G., BAGATELLA, M., DONADIO, M. & 5 others (1987). Mitomycin C, vinblastine and cisplatin. An active regimen for advanced non-small cell lung cancer. *Br. J. Cancer*, **56**, 475.

GRALLA, R.J., CASPER, E.S., KELSEN, D.P. & 5 others (1981). Cisplatin and vindesine combination chemotherapy for advanced carcinoma of the lung: a randomised trial investigating two dosage schedules. *Ann. Intern. Med.*, **95**, 414.

GRALLA, R.J. & KRIS, M.G. (1988). Chemotherapy in non-small cell lung cancer: results of recent trials. *Semin. Oncol.*, **15**, 2

HOFFMAN, P.C., BITRAN, J.D. & GOLOMB, H.M. (1983). Chemotherapy of metastatic non-small cell bronchogenic carcinoma. *Semin. Oncol.*, **10**, 111.

LAD, T.E., NELSON, R.B., DICKAMP U. & 8 others (1981). Immediate versus postponed combination chemotherapy (CAMP) for unresectable non-small cell lung cancer: a randomised trial. *Cancer Treat. Rep.*, **65**, 973.

LAING, A.H., BERRY, R.J., NEWMAN, C.R. & PETO, J. (1975). Treatment of inoperable carcinoma of bronchus. *Lancet*, **ii**, 1161.

MACKILLOP, W.J., O'SULLIVAN, B. & WARD, G.K. (1987). Non-small cell lung cancer: how oncologists want to be treated. *Int. J. Radiat. Oncol. Biol. Phys.*, **13**, 929.

MILLER, A.B., HOOGSTRATEN, B., STAQUET, M. & WINKLER, A. (1981). Reporting results of cancer treatment. *Cancer*, **47**, 207.

RAPP, E., PATER, J.L., WILLAN, A. & 12 others (1988). Chemotherapy can prolong survival in patients with advanced non-small cell lung cancer – report of a Canadian multicenter radomised trial. *J. Clin. Oncol.*, **6**, 633.

SCULIER, J.-P. & KLATERSKY, J. (1984). Progress in chemotherapy of non-small cell lung cancer. *Eur. J. Cancer Clin. Oncol.*, **20**, 1329.

WILLIAMS, C.J., WOODS, R., LEVI, J. & PAGE, J. (1988). Chemotherapy for non-small cell lung cancer: a randomised trial of cisplatin/vindesine vs no chemotherapy. Perugia International Cancer Conference 1: Chemotherapy of non-small cell lung cancer–current status. Perugia, 17–18 June, 1988, p. 43 (Abstract).